# Delayed onset of autoreactive antibody production and M2-skewed macrophages contribute to improved survival of TACI deficient MRL-Fas/Lpr mouse

**DOI:** 10.1038/s41598-018-19827-8

**Published:** 2018-01-22

**Authors:** Lunhua Liu, Windy Rose Allman, Adam Steven Coleman, Kazuyo Takeda, Tsai-Lien Lin, Mustafa Akkoyunlu

**Affiliations:** 1 Laboratory of Bacterial Polysaccharides, Division of Bacterial Parasitic and Allergenic Products, Silver Spring, MD 20993 United States of America; 2 Microscopy and Imaging Core Facility, Division of Viral Products, Silver Spring, MD 20993 United States of America; 3 Vaccine Evaluation Branch, Division of Biostatistics, Center for Biologics Evaluation and Research, Food and Drug Administration, Silver Spring, MD 20993 United States of America

## Abstract

Anti-B cell activating factor belonging to TNF-family (BAFF) antibody therapy is indicated for the treatment of patients with active systemic lupus erythematosus (SLE). We hypothesized that the BAFF receptor, transmembrane activator and calcium-modulator and cyclophilin interactor (TACI) may be responsible for the generation of antibody secreting plasma cells in SLE. To test this hypothesis, we generated TACI deficient MRL-Fas/Lpr (LPR-TACI−/−) mouse. TACI deficiency resulted in improved survival of MRL-Fas/Lpr mice and delayed production of anti-dsDNA and anti-SAM/RNP antibodies. There was also a delay in the onset of proteinuria and the accumulation of IgG and inflammatory macrophages (Mϕs) in the glomeruli of young LPR-TACI−/− mice compared to wild-type mice. Underscoring the role of TACI in influencing Mϕ phenotype, the transfer of Mϕs from 12-week-old LPR-TACI−/− mice to age-matched sick wild-type animals led to a decrease in proteinuria and improvement in kidney pathology. The fact that, in LPR-TACI−/− mouse a more pronounced delay was in IgM and IgG3 autoreactive antibody isotypes and the kinetics of follicular helper T (Tfh) cell-development was comparable between the littermates suggest a role for TACI in T cell-independent autoantibody production in MRL-Fas/Lpr mouse prior to the onset of T cell-dependent antibody production.

## Introduction

Systemic lupus erythematosus (SLE) is an autoimmune disease characterized by autoantibody overproduction because of dysregulated innate and adaptive immune function^1,2^. Although conventional agents such as corticosteroids, antimalarial drugs, and other immunosuppressive therapies provide enhanced survival in patients with SLE, their use is associated with considerable toxicity and a large proportion of patients remain refractory to the treatment^2–5^. Shortly after the discovery of B cell activating factor belonging to TNF-family (BAFF) as a cytokine critically important in B cell survival^6^, an association between SLE and elevated circulating BAFF was established in mouse models^7^ and in SLE patients^8,9^. Underscoring the role of BAFF in SLE, depletion or ablation of BAFF in lupus prone mice resulted in reduced disease severity and mortality^10,11^. In contrast to BAFF, both positive and negative correlations were observed between serum levels of the related cytokine, a proliferation-inducing ligand (APRIL), and disease severity^12–18^. Investigation of APRIL’s contribution to SLE in mouse models also produced inconclusive results. For example, neither the transgenic mice overexpressing APRIL nor the SLE-prone NZM mice with disrupted APRIL gene manifested a change in autoimmune phenotype^19,20^. However, ablation of APRIL with selective monoclonal antibody resulted in delayed SLE development in NZM mice and APRIL disruption in Nba2.Yaa mice improved SLE disease with reduced pathogenic antibody production and glomerulonephritis development^21,22^. Recognition of the importance of BAFF in the generation and/or sustainment of autoreactive B cells led to BAFF inhibition strategies as a treatment for SLE^23^. These efforts resulted in the approval of Belimumab (a neutralizing antibody against BAFF) in 2011 for the treatment of SLE patients with active, autoantibody-positive disease^24,25^.

BAFF binds to BAFF receptor (BAFFR), transmembrane activator and calcium modulator and cyclophilin ligand interactor (TACI), and B-cell maturation antigen (BCMA), while APRIL only exhibits high affinity to TACI and BCMA^26^. Although the alleviation of disease symptoms with anti-BAFF therapy underscored the importance of BAFF in SLE pathogenesis, the contribution of individual receptors to BAFF action is poorly understood. Compared to healthy subjects, B cells from patients with SLE have lower or similar BAFFR expression. The expression of BCMA and TACI in the same patients have been reported as higher, lower or the same as healthy individuals^27–31^. To address the role of these receptors in SLE, several different lupus mouse strains with deficiencies in individual or combination of receptors were generated. The deletion of BAFFR alone or a loss of function mutation of BAFFR was not beneficial in any tested mouse strain^32,33^. Deletion of BCMA in B6 LPR mice resulted in increased lethality, while the course and severity of disease in BCMA-deficient NZM mice was not different than the wild-type mice^32,34^. Attempts to study the role of TACI in SLE mouse models have been less conclusive. For example, TACI deficient NZM mice survival was not affected despite manifesting with increased renal pathology^32^. On the other hand, using a bone marrow chimera strategy, Figgett *et al*. have shown that TACI deficiency prevented the onset of autoimmune manifestations in BAFF transgenic mice^35^. TACI deficiency was also beneficial in NZM mice deficient in both TACI and BAFFR^36^. Finally, a more recent study reported decreased mortality accompanied by lower levels of autoantibodies and glomerulonephritis in TACI deficient male Nba2.Yaa mice^22^.

In light of these diverse outcomes regarding the role of TACI in SLE, we sought to investigate the contribution of TACI in MRL-Fas/Lpr mice, which develop SLE like autoimmune manifestation as a result of a spontaneous mutation in the *fas* gene^37^. We observed that TACI-deficient MRL-Fas/Lpr mice (LPR-TACI−/−) survived significantly longer than the wild-type mice (LPR-TACI+/+). The delayed mortality was accompanied by a delay in autoreactive antibody development and the onset of lupus nephritis. In SLE, the inflammation initiated by the deposition of immune complexes in glomeruli leads to the accumulation of M1-polarized macrophages (Mϕs), which augment kidney pathology^38,39^. Conversely, the M2-polarized Mϕs are believed to maintain a protective anti-inflammatory environment. The kidneys of 12-week-old LPR-TACI−/− mice were populated with M2-like Mϕs, while the age matched WT mice kidneys were infiltrated with M1-like Mϕs. The M2-phenotype of Mϕs isolated from LPR-TACI−/− mice is consistent with our recent discovery of a role for TACI in mediating M1 polarizing signals^40^. The fact that spontaneous accumulation of germinal center (GC) B cells, plasma cells, follicular T helper (Tfh) cells as well as extrafollicular Tfh (Thef) cells in LPR-TACI−/− mice was not significantly different than the wild-type mice suggested that TACI deficiency does not impact the development T cell-dependent autoreactive antibodies. Instead, TACI deficiency is likely responsible for the delay in disease onset by preventing T cell-independent autoreactive antibody production and by maintaining an anti-inflammatory environment in kidneys owing to M2 phenotype of TACI deficient Mϕs.

## Results

### TACI deficient MRL-Fas/Lpr mice manifest decreased lymphadenopathy and prolonged survival

**Figure 1:**
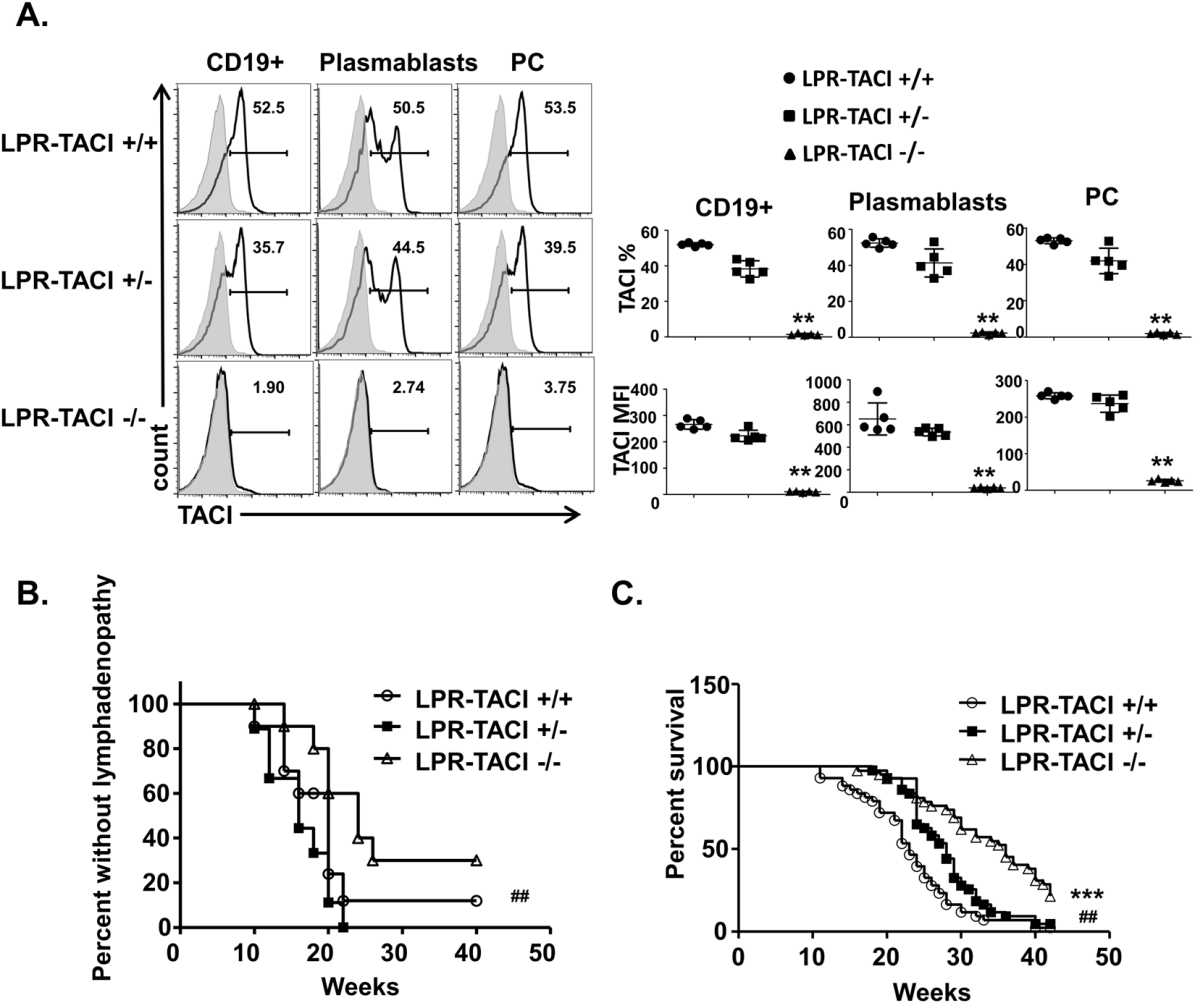
TACI deficient MRL-Fas/Lpr mice manifest decreased lymphadenopathy and prolonged survival. (**A**) Representative histograms as well as mean percentages and mean fluorescence intensities (MFI) of TACI expression on splenic B cells (CD19+), plasmablasts (CD19 + CD138+) and plasma cells (PC) (CD19 − CD138+) of 6 to 8-week-old LPR-TACI+/+, LPR-TACI+/− and LPR-TACI+/− mice are shown. The data shown are from 5 female mice per group. (**B**) Lymphadenopathy assessed as enlarged lymph nodes over 40 weeks. LPR-TACI−/− mice had reduced lymphadenopathy compared to LPR-TACI+/+ and LPR-TACI+/− mice. (**C**) Percent survival rates over 42 months. The cumulative survival of female LPR-TACI+/+, LPR-TACI+/−, and LPR-TACI−/− mice was monitored daily for 42 weeks. TACI deficient MRL-Fas/Lpr mice exhibited significantly better survival curve compared with the two other groups. **p < 0.01 and ***p < 0.001 indicate statistical differences between LPR-TACI+/+ and LPR-TACI−/− mice. ^##^p < 0.01 indicates statistical difference between LPR-TACI+/− and LPR-TACI−/− mice.

Similar to patients with SLE, MRL-Fas/Lpr mice present with a complex autoimmune inflammatory manifestations, including nephritis, arthritis, lymphadenopathy, and splenomegaly^37^. To examine the impact of TACI on the phenotype of MRL-Fas/Lpr mice, we generated TACI-deficient mice on an MRL-Fas/Lpr background (LPR-TACI−/−). TACI deficiency was confirmed by evaluating surface TACI protein expression on total B cells (CD19+), plasmablasts (CD19 + CD138+), and plasma cells (CD19 − CD138+) in spleens of LPR-TACI+/+, LPR-TACI+/−, and LPR-TACI−/− mice (Fig. 1A). As has been shown previously in TACI deficient mice on C57BL/6 background^41^, TACI deficient lupus prone mice on MRL-Fas/Lpr background also had an elevated number of splenic B cells at 6 weeks of age as compared to wild-type MRL-Fas/Lpr mice (Supplemental Fig. 1A). However, this difference vanished after 8 weeks of age. The expression of TACI on total B cells of LPR-TACI+/+ mice did not significantly change between the ages of 6 weeks to 12 weeks (Supplemental Fig. 2A,B). There was however, a gradual and significant decrease of TACI expression on B cells of LPR-TACI+/− mice starting from 8 weeks of age (Supplemental Fig. 2A,B). A similar decrease was seen in plasmablasts and plasma cells of LPR-TACI+/− mice. We have previously shown that MRL strains express reduced levels of BAFFR on B cells compared to Balb/c mice, a likely consequence of the Pro44Ser mutation in BAFFR gene, TNFRSF13C^42^. We found similarly modest expression of BAFFR on TACI-LPR−/− and TACI-LPR+/− mice B cells (Supplemental Fig. 2A,C). Moreover, TACI depletion did not significantly alter the expression of BAFFR and BCMA on splenic B cells, plasmablasts, or plasma cells in these mouse strains (Supplemental Fig. 2A,C,D).

Next, we monitored the health and survival of LPR-TACI−/− mice, as well as their LPR-TACI+/− and LPR-TACI+/+ littermates for a period of 42 weeks. All strains appeared healthy and fertile at an early age (0–8 weeks). As in human SLE, T and B cell-hyperproliferation causes lymphadenopathy in MRL-Fas/Lpr mice^37^. Like MRL-Fas/Lpr mice, LPR-TACI+/+ and LPR-TACI+/− mice rapidly developed lymphadenopathy (Fig. 1B). However, lymphadenopathy development was delayed in LPR-TACI−/− mice compared to the control littermates although the percent of mice with lymphadenopathy was statistically significantly different only between LPR-TACI+/− and LPR-TACI+/+ mice. More importantly, compared to LPR-TACI+/+ and LPR-TACI+/− mice, both of which showed similar survival curves (50% mortality around age 24 weeks in both groups), LPR-TACI−/− mice had significantly improved survival (>80% survival at age 24 weeks) (Fig. 1C). Thus, TACI deficiency delayed clinical manifestation of lupus disease and extended the life span of lupus mice.

### TACI deficiency slows down the anti-dsDNA, RNP/Sm and anti-nuclear antibody responses in MRL-Fas/Lpr mice

**Figure 2:**
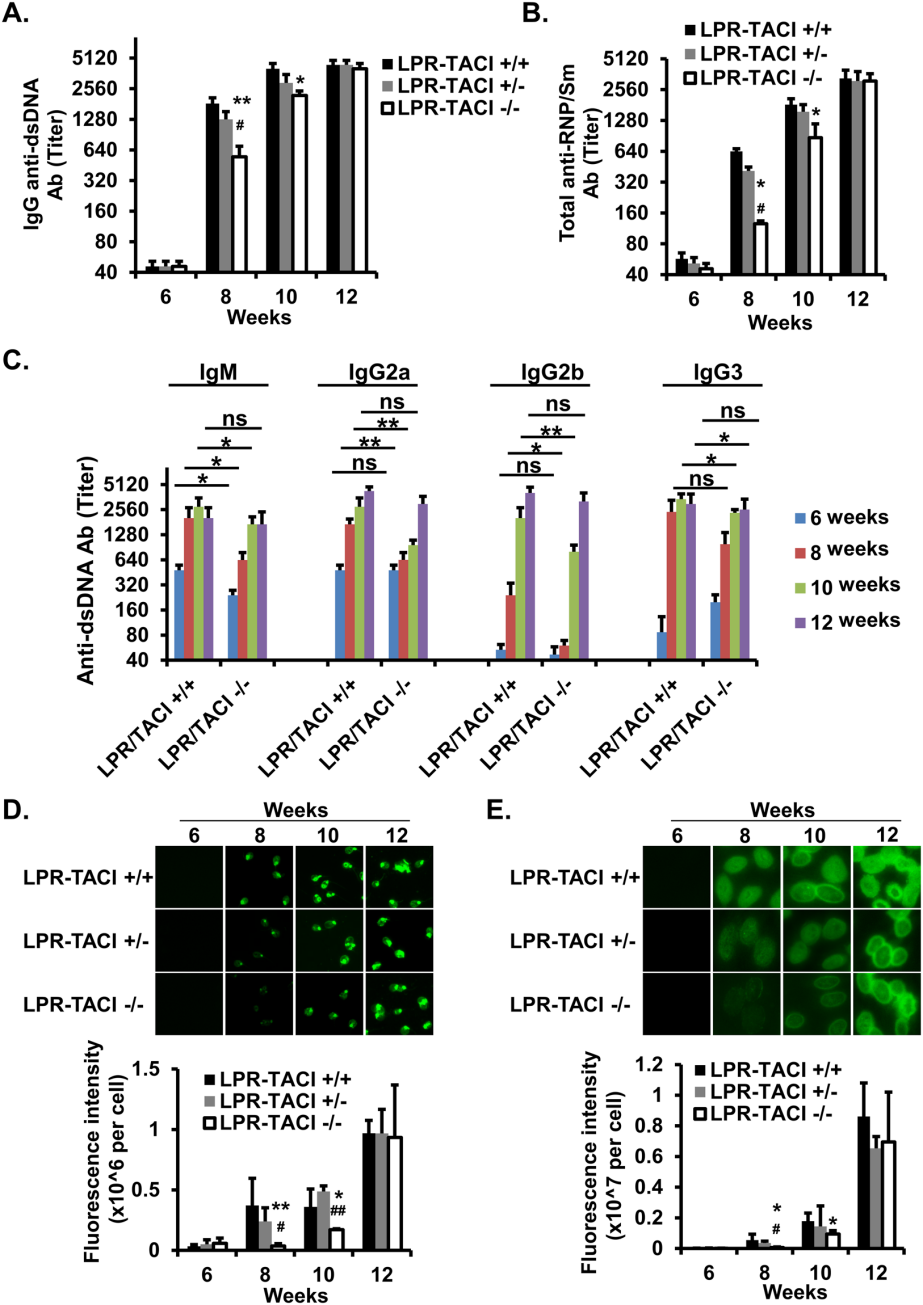
Serum autoreactive antibody development is delayed in TACI deficient MRL-Fas/Lpr mice. Titers of serum anti-dsDNA total IgG antibodies (**A**), anti-RNP/Sm antibodies (**B**), and anti-dsDNA IgM, IgG2a, IgG2b, IgG3 antibodies isotypes (**C**) were determined by ELISA. Titer is defined by the serum dilution exhibiting an OD reading 2 times higher than background. Mean titers ± SD from 5 to 7 mice in each group were plotted. Anti-dsDNA antibodies (**D**) and Antinuclear antibodies (**E**) were assessed by indirect immunofluorescence measurement. Serum anti-dsDNA antibodies were detected using *C. luciliae* slides and ANAs were detected by HEp-2 slides. The fluorescence intensity of single cell was quantified with ImageJ and plotted. The data shown are from 5 female mice per age group. *p < 0.05 and **p < 0.01 indicate statistical differences between LPR-TACI+/+ and LPR-TACI−/− mice. ^#^p < 0.05 and ^##^p < 0.01 indicate statistical differences between LPR-TACI+/− and LPR-TACI−/− mice.

Elevation in serum autoreactive antibodies is the hallmark characteristic of SLE^41,43^. Since TACI is critically important in the development and maintenance of plasma cells^44–47^, we expected TACI deficient MRL-Fas/Lpr mice to mount a lesser antibody response against nuclear material. From 6 to 12 weeks of age, serum levels of anti-dsDNA IgG and anti-RNP/Sm total antibodies increased progressively with age in all three groups (Fig. 2A,B). However, compared to LPR-TACI+/+ controls, LPR-TACI−/− mice had significantly lower levels of circulating dsDNA and RNP/Sm autoantibodies at the 8 and 10-week time points (Fig. 2A,B). There was also a significant difference in the levels of serum anti-dsDNA IgG2a, IgG2b, and IgG3 isotypes between LPR-TACI+/+ and LPR-TACI−/− mice at ages 8 and 10 weeks (Fig. 2C). The different in anti-dsDNA IgM antibody titers between LPR-TACI+/+ and LPR-TACI−/− mice started at 6 weeks and persisted thereafter on weeks 8 and 10 weeks. This differences in all antibody isotypes vanished by 12 weeks of age. The reduced serum anti-dsDNA and anti-RNP/Sm autoantibodies in 8 and 10-week-old LPR-TACI−/− mice were further confirmed with indirect fluorescence microscopy using C. *luciliae* and HEp-2 diagnostic slides (Fig. 2D,E). Thus, in TACI deficient MRL-Fas/Lpr mice, serum anti-dsDNA, RNP/Sm, and antinuclear autoantibody (ANA) development is significantly delayed.

### TACI deficiency delays the onset of lupus nephritis

**Figure 3:**
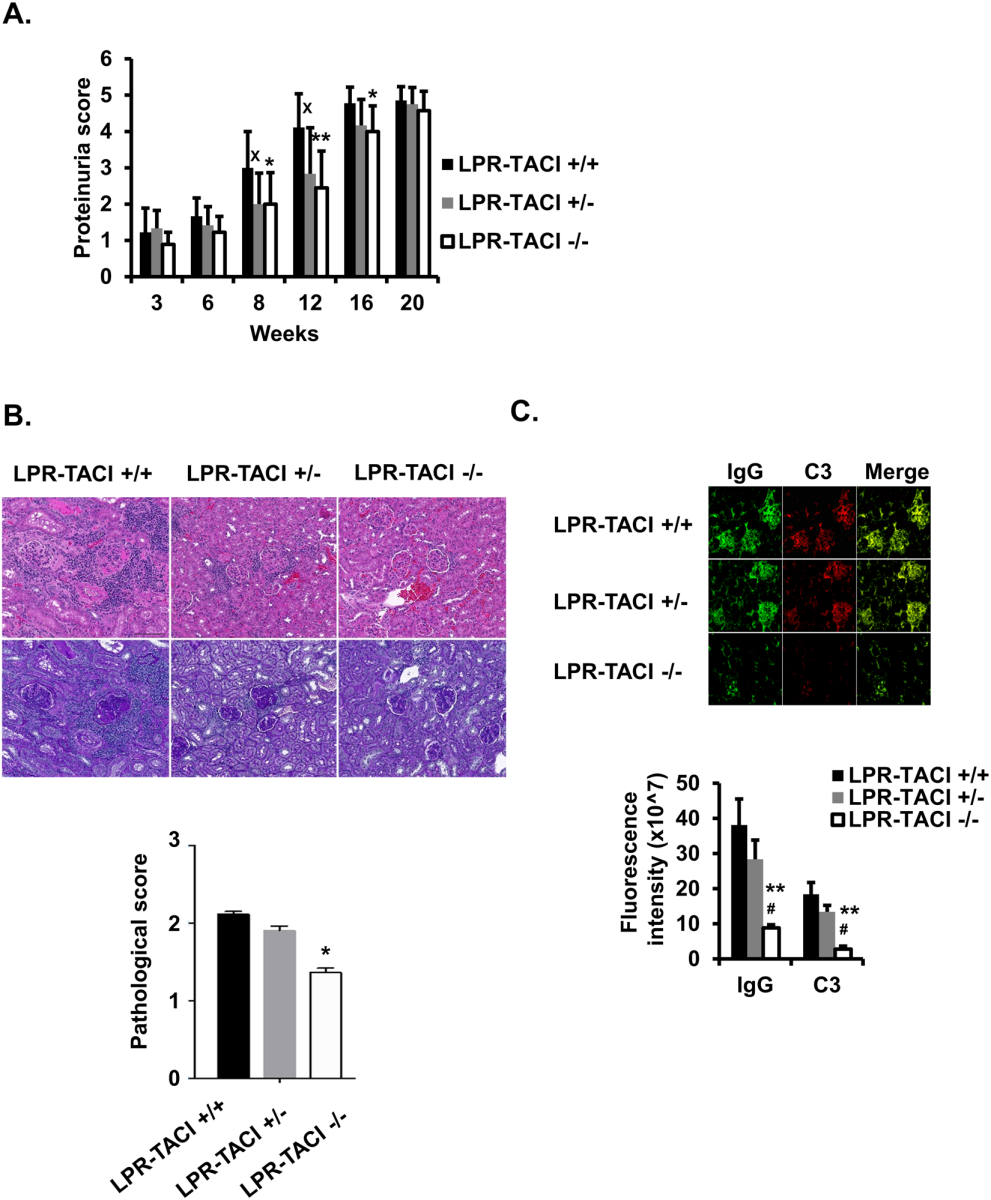
The delayed kidney pathology is accompanied by less IgG and C3 deposition in TACI deficient MRL-Fas/Lpr mice. (**A**) TACI deficient MRL-Fas/Lpr mice showed a delayed proteinuria onset. Mean ± SD of proteinuria scores are plotted. The data shown are from 3 to 20-week-old mice. Each group contained 7 to 9 female mice. (**B**) Assessment of kidney pathology. Upper panel shows a representative image of H&E (upper row) and PAS (bottom row) stained kidney specimens from 14-week-old mice. Mesangial cell proliferation and increase of mesangial matrix with inflammatory cell infiltration were more frequently in LPR-TACI+/+ and LPR-TACI+/− kidney tissue samples than TACI-LPR−/− samples. Glomerular changes were scored on a scale of 0 (no pathology) to 3 (severe pathology). (**C**) SLE related kidney pathology is evaluated by assessment of renal C3 and IgG deposition. Representative immunofluorescence images of IgG (green) and C3 (red) immune deposits in the glomeruli of 12 weeks old mice are shown. Fluorescence intensity ± SD from 5 mice were quantified by imageJ and plotted. x p < 0.05 indicates statistical differences between LPR-TACI+/+ and LPR-TACI+/− mice. *p < 0.05 and **p < 0.01 indicate statistical differences between LPR-TACI+/+ and LPR-TACI−/− mice. ^#^Indicates p < 0.05 for statistical difference between LPR-TACI+/− and LPR-TACI−/− mice.

The elevation in systemic autoreactive antibodies and the deposition of immune complexes in glomeruli is the primary cause of kidney failure in SLE, the most common cause of mortality in patients with SLE^48^. To assess kidney disease and pathology, we measured proteinuria and performed histopathological examinations in kidneys from LPR-TACI+/+, LPR-TACI+/−, and LPR-TACI−/− mice. The LPR-TACI+/+ group showed higher levels of proteinuria at the beginning of 8 weeks, indicating the early onset of kidney disease (Fig. 3A). As expected, proteinuria levels in LPR-TACI+/+ mice progressively increased from 8 to 16 weeks of age. By comparison, although proteinuria levels also rose with age, both LPR-TACI+/− and LPR-TACI−/− mice manifested milder proteinuria than age matched LPR-TACI+/+ mice until 20 weeks of age, when the difference between strains vanished (Fig. 3A). We chose a 14-week time point to evaluate histopathological changes among mouse strains when the differences in proteinuria were most significant among wild-type and TACI-deficient mice. Although glomerular pathology was not entirely prevented, the glomerular histopathological changes of LPR-TACI−/− mice were significantly reduced compared with age-matched LPR-TACI+/+ and LPR-TACI+/− mice (Fig. 3B).

The deposition of IgG and C3 in the glomeruli is one of the prominent features of lupus nephritis and is the principal underlying cause for kidney damage in MRL-Fas/Lpr mice^49,50^. We detected IgG and C3 deposits within the peripheral glomerular capillary loops and the mesangium in 12-week-old LPR-TACI+/+ and LPR-TACI+/− mice (Fig. 3C). In contrast, significantly less deposition of IgG and C3 was detected in age-matched LPR-TACI−/− littermates. Pathological analyses indicated that the delayed autoreactive antibody production is likely responsible for the late onset of lupus nephritis in TACI deficient MRL-Fas/Lpr mice.

### M2 skewed LPR-TACI−/− Mϕs contribute to the prevention of kidney damage in TACI deficient MRL-Fas/Lpr mice

**Figure 4:**
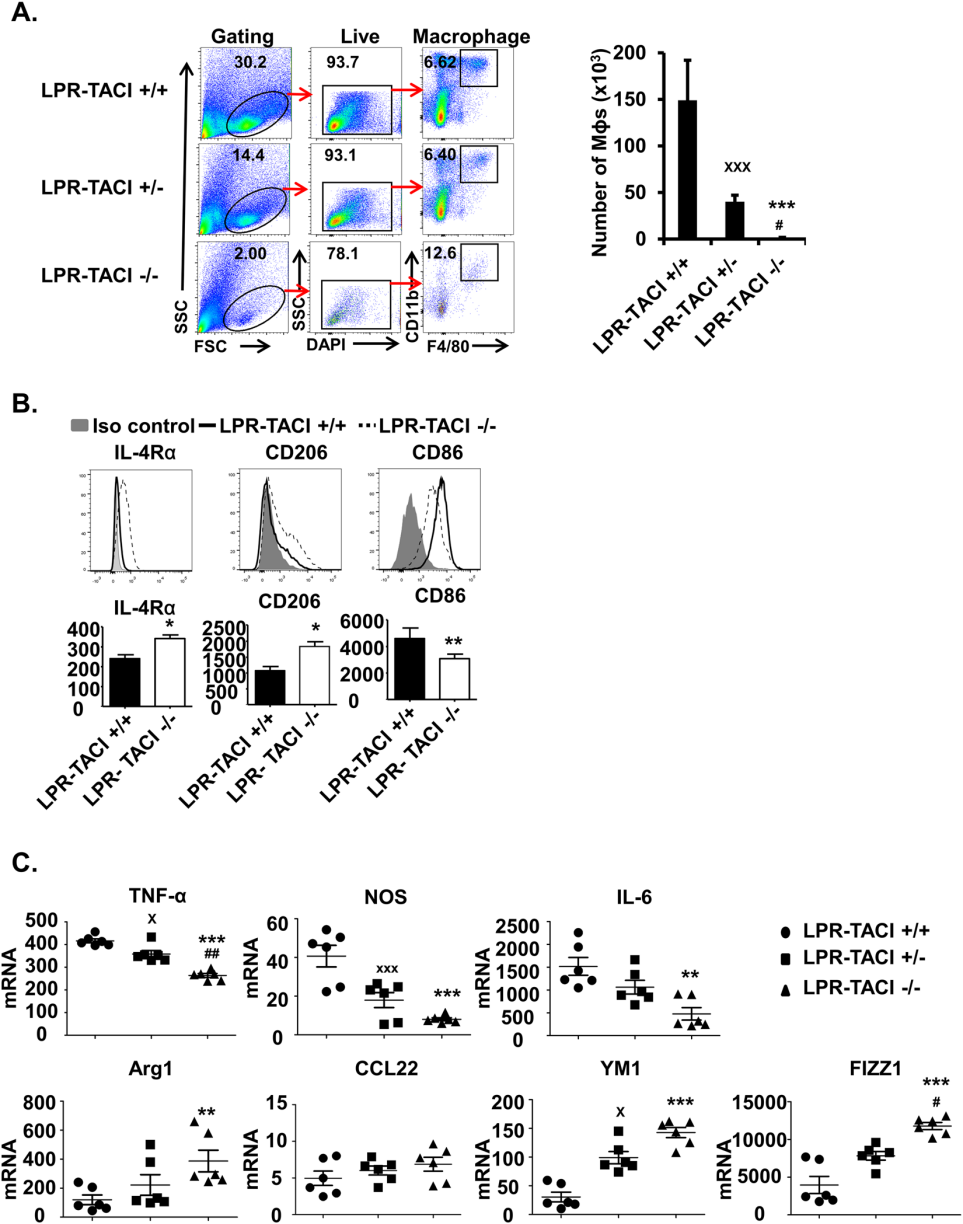
Renal Mϕs from TACI deficient MRL-Fas/Lpr mice exhibit M2-skewed phenotype. (**A**) Isolation of Mϕs from kidneys. Kidneys from 12-weeks old mice were first minced and then digested with collagenase. The fraction containing immune cells were isolated after Percoll gradient centrifugation. Dead cells were excluded firstly, the Mϕs were gated as F4/80 and CD11b positive cells and quantified. (**B**) Phenotypes of Mϕs isolated from kidneys were assessed in flow cytometry. The expression of the M2 markers, IL-4Rα, CD206 and M1 marker CD86 in renal Mϕs was measured by FACS. A representative histogram for each marker is shown. Mean fluorescence intensities of IL-4Rα, CD206 and CD86 staining from 5 female mice per group were plotted. (**C**) Renal Mϕs were sorted from 12-weeks old mice. Gene expression of M1 and M2-associated markers in the sorted cells was assessed by Q-PCR. ^x^p < 0.05 and ^xxx^p < 0.001 indicate statistical differences between LPR-TACI+/+ and LPR-TACI+/− mice. *p < 0.05, **p < 0.01 and ***p < 0.001 indicate statistical differences between LPR-TACI+/+ and LPR-TACI−/− mice. ^#^p < 0.05 and ^##^p < 0.01 indicate statistical differences between LPR-TACI+/− and LPR-TACI−/− mice.

Increased accumulation of activated (M1-like) Mϕs in the kidney coincides with the onset and progression of SLE disease. Furthermore, the persistence of activated Mϕs is highly associated with poor clinical outcomes^38,39,51,52^. Having established a prominent difference between the mouse strains in both kidney disease and pathology at 12 weeks of age, we focused our attention in characterizing renal Mϕs. The analysis of the renal Mϕ population in 12-week-old mice indicated that TACI deficient lupus mice Mϕ-infiltration remained significantly less than LPR-TACI+/+ and LPR-TACI+/− mice (Fig. 4A). Moreover, unlike renal Mϕs from LPR-TACI+/+ mice, which expressed elevated levels of the M1-Mϕ surface marker CD86, LPR-TACI−/− mice Mϕs expressed higher M2-Mϕ markers, such as mannose receptor (CD206) and IL4Rα as detected in flow cytometry analysis (Fig. 4B). To further characterize their phenotypes, we sorted renal Mϕs and quantified the representative genes that define M1- and M2-Mϕs in real-time PCR analyses. Purified renal Mϕs as well as peritoneal Mϕs from LPR-TACI−/− mice exhibited decreased TNF-α, NOS and IL-6 but enhanced Arg1, YM1 and FIZZ1 mRNA levels (Fig. 4C, Supplemental Fig. 3A). These gene expression analyses strongly indicated that reduced kidney pathology in LPR-TACI−/− mice was accompanied by M2-skewed renal Mϕs, while kidneys of wild-type littermates were infiltrated with the inflammatory M1-Mϕs. We have previously shown that BAFF and APRIL induce M1-polarization of Mϕs through TACI^40^. Therefore, in LPR-TACI−/− mice, in addition to limited immune complex deposition, renal Mϕs may be protected from inflammatory-Mϕ polarization signals mediated by TACI. However, LPR-TACI−/− mice Mϕs could not sustain their M2-phenotype at later time points when the TACI deficient lupus mice proteinuria levels reached levels comparable to those of wild-type littermates. Kidneys of 26-week-old LPR-TACI−/− mice harbored similar frequencies of Mϕs as littermates and the expression levels of M1 and M2 marker genes in Mϕs harvested from kidneys were not significant different between the littermates (Supplemental Fig. 3B,C).

**Figure 5:**
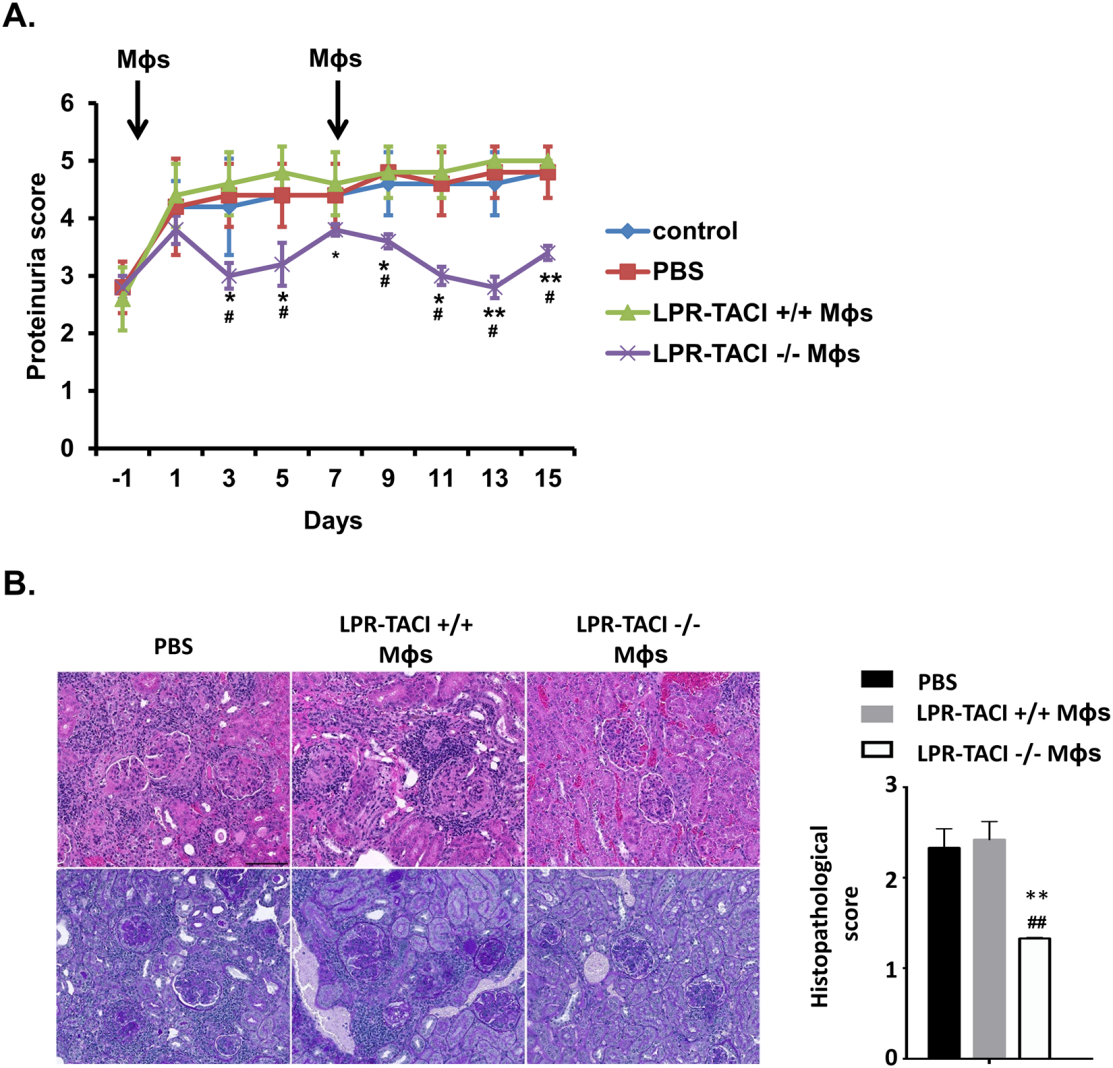
Adoptively transferred TACI deficient Mϕs alleviate glomerulonephritis development in MRL-Fas/Lpr mice. (**A**) Proteinuria measurement in MRL-Fas/Lpr mice adoptively transferred with LPR-TACI+/+ or LPR-TACI−/− Mϕs. Macrophages isolated from 12-week old LPR-TACI+/+ or LPR-TACI−/− mice were i.v. injected into 10 weeks-old MRL-Fas/Lpr mice on indicated time points. Control MRL-Fas/Lpr mice received equal volume of PBS. Proteinuria measured on every two days from 5 mice from each group and mean proteinuria score ± SD were plotted. *p < 0.05 and **p < 0.01 indicates statistical differences between LPR-TACI+/+ Mϕs transferred groups and LPR-TACI−/− Mϕs transferred groups. ^#^p < 0.05 indicates statistical difference between PBS injected and LPR-TACI−/− Mϕs transferred groups. (**B**) Representative images of H&E (upper row) and PAS (bottom row) stained kidney specimens. Glomerular changes, inflammatory cell infiltration and interstitial fibrosis were semi-quantitatively scored on a scale of 0 (no pathology) to 3 (severe pathology). **p < 0.01 indicates statistical difference between LPR-TACI+/+ Mϕ injected and LPR-TACI−/− Mϕ transferred groups. ^##^p < 0.01 indicates statistical difference between PBS injected and LPR-TACI−/− Mϕ transferred groups.

To further interrogate the significance of TACI deficient Mϕs in the prevention of kidney dysfunction, we performed adoptive transfer experiments. Ten-week-old MRL-Fas/Lpr mice with moderate proteinuria were transferred with Mϕs isolated from kidneys of 12-week-old LPR-TACI+/+ or LPR-TACI−/− mice. Mice injected with LPR-TACI+/+ Mϕs manifested slightly elevated proteinuria as compared to PBS injected or naïve mice until 7 days after the first injection (Fig. 5A). In contrast, Mϕs from TACI-LPR−/− mice initiated a significant reduction in proteinuria at day 3 after a modest increase 1 day after the injection of Mϕs (Fig. 5A). Proteinuria in mice injected with LPR-TACI−/− Mϕs remained significantly lower than all other mouse groups until day 7, although there was an increase-trend after day 3. A second injection of renal Mϕs from LPR-TACI−/− mice triggered further decrease in proteinuria in LPR-TACI+/+ mice for another 8 days. Progression of proteinuria continued in all other mouse groups throughout the 15-day follow up duration. In concert with the improvements in proteinuria levels, kidney sections from LPR-TACI−/− Mϕ-injected mice revealed significantly less histopathological changes as compared to those of LPR-TACI+/+ Mϕ-injected or PBS-injected mice (Fig. 5B). Thus, the improvements in kidney disease and pathology elicited by the transfer of TACI deficient Mϕs highlight the role Mϕs play in orchestrating the outcome of renal inflammatory changes in SLE.

### T helper cell response associated with autoreactive antibody production is modestly altered in TACI deficient MRL-Fas/Lpr mice

Since TACI promotes the differentiation of B cells to plasma cells^45–47,53^, we next assessed the frequencies of these cells to determine whether the delayed anti-dsDNA and RNP autoreactive antibody production is associated with altered plasma cell development. As expected, in addition to total B cell numbers, plasmablasts and plasma cells populated spleens of wild-type MRL-Fas/Lpr lupus mice with age significantly more than Balb/c mice or control MRL mice (Supplemental Fig. 4A–D). Following the same pattern, the number of splenic plasmablasts and plasma cells also increased in LPR-TACI−/− mice as well as in LPR-TACI+/− and LPR-TACI+/+ littermates with age (Supplemental Fig. 4E,F).

**Figure 6:**
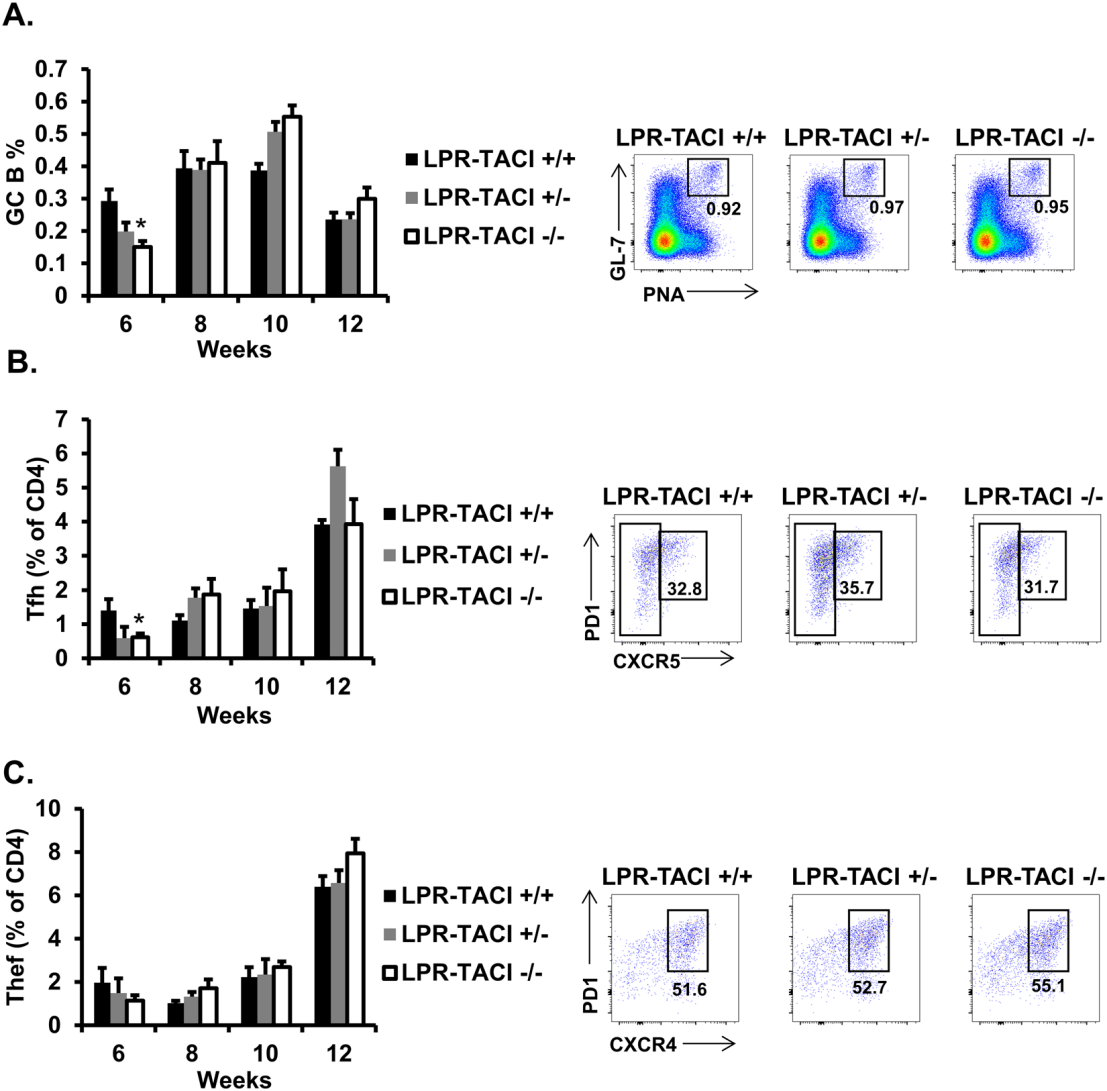
Spontaneous GC, Tfh and Thef accumulations were not altered in TACI deficient MRL-Fas/Lpr mice. Percentages of CD19 + CD3-GL7 + PNA + GC B cells (**A**), CXCR4-CXCR5+ PD1+ PSGL1loCD62L-CD44+ Tfh cells (**B**), CXCR4+ CXCR5-PD1+ PSGL1loCD62L-CD44+ Thef cells (**C**) in spleens of 6 to 12 weeks old LPR-TACI+/+, LPR-TACI+/− and LPR-TACI−/− mice were quantified in flow cytometry. Mean percentage ± SD from 4 to 6 mice in each group were plotted. *p < 0.05 indicates statistical difference between LPR-TACI+/+ and LPR-TACI−/− mice.

In MRL-Fas/Lpr mice, T cell-dependent autoreactive antibody formation, isotype switching, and affinity maturation take place in spontaneously formed GCs^54^. The effector T helper cell subsets, Tfh cells, and the extrafollicular helper T cells (Thef) all contribute to the GC-dependent and extrafollicular autoreactive antibody production^55–58^. By using GL-7 and PNA markers (Supplemental Fig. 5A), we confirmed spontaneous splenic GC formation in the wild-type MRL-Fas/Lpr mice starting from 8 weeks of age (Supplemental Fig. 5C). Interestingly, the percent of GC B cells decreased after peaking at 10 weeks to lower than that of 8-week-old MRL-Fas/Lpr mice. The control MRL mice GC formation followed a similar pattern, albeit to a lower degree. Spontaneous Tfh and Thef development also started at 6 weeks of age in MRL-Fas/Lpr mice and gradually increased thereafter with age (Supplemental Fig. 5B,D,E). The increase in MRL mice Tfh cell numbers started 4 weeks later than those of lupus mice, whereas MRL and lupus mice Thef cell numbers were comparable at all time points. To assess whether the delay in autoreactive antibody formation in TACI deficient lupus mice is associated with a change in the kinetics of GC formation, we compared splenic GC B cell frequencies in LPR-TACI−/− mice and littermates. The percentage of GC B cells increased until 10 weeks of age in all strains (Fig. 6A). As in MRL-Fas/Lpr mice (Supplemental Fig. 5C), GC B cell numbers decreased in all three littermates after 10 weeks of age (Fig. 6A). The percentage of GC B cells was significantly lower in LPR-TACI−/− mice than LPR-TACI+/+ mice at 6 weeks but this difference vanished at later time points. A similar difference was observed at 6 weeks of age for Tfh cell frequencies between LPR-TACI−/− and LPR-TACI+/+ mice, which also disappeared as Tfh cell percentages gradually increased with age in all littermates (Fig. 6B, See Supplemental Fig. 5B for gating strategy). Thef cells also increased with age with a difference among the littermates (Fig. 6C, See supplemental Fig. 5B for gating strategy). Overall, the analyses of the cellular components that participate in T cell-dependent antibody formation indicated that other than the differences at early time point (6 weeks) a significant difference was not detected in the expansion of GC B cell and Tfh cells between TACI deficient and TACI sufficient lupus mice.

## Discussion

The approval of the BAFF antagonist Belimumab for the treatment of SLE spotlighted the critical role of BAFF receptors in the pathogenesis of disease. Whether TACI exacerbates or attenuates pathological manifestations of SLE remains an unresolved question. The increase in B cell numbers^41,43^ and autoantibody titers^59^ in TACI deficient C57BL/6 mice predict aggravated SLE development in the absence of TACI on SLE background. On the other hand, impaired antibody responses to T cell-independent antigens^41,43^ as well as decreased plasma cell generation^47^, a consequence of blunted response to cytokines BAFF and APRIL^45^ in TACI knock-out C57BL/6 mice, would predict attenuated SLE manifestations in TACI deficient lupus mice. Using MRL-Fas/Lpr mice, we found a significantly delayed onset of disease and improved survival when TACI is absent in a mouse model of lupus. In addition to detecting the previously observed decrease in autoantibody production in TACI deficient SLE mouse models^22,35^, we discovered a novel mechanism of protection associated with TACI deficiency whereby M2-skewed Mϕs contribute to the alleviation of renal inflammation.

In contrast to TACI deficient NZM mice, which exhibited a clinical course identical to the wild-type mice^32^, TACI deficiency in MRL-Fas/Lpr mice led to reduced lupus activity, including diminished kidney pathology in our studies. In BAFF transgenic C57BL/6 mice, the transfer of TACI deficient bone marrow cells fully protected the animals from autoantibody production and SLE pathogenesis^35^. We did not observe a complete prevention from disease in MRL-Fas/Lpr mice when TACI is absent. Instead, TACI depletion reduced the amount of autoreactive antibodies and attenuated the disease symptoms only at the young age. The discrepancy in disease outcomes among different mouse models could be partially explained by the degree of dependency to BAFF for the autoreactive antibody production in each model. In NZM mice, autoreactive B cells could survive and secrete antibodies in the complete absence of BAFF^11^. Similarly, BAFF blockade using a soluble TACI-expressing adenovirus vector (adTACI) or TACI-Ig had little effect on autoantibody production in NZB/W F1 mice^10,60^. In contrast, the same adTACI construct could significantly decrease the autoantibody production in MRL Fas/Lpr mice^60^. The relative independence of NZB/W F1 mice from BAFF for the lupus phenotype can be explained by the fact that NZB/W F1 mice SLE pathogenesis is linked to major histocompatibility complex class II (MHCII) locus and pathogenic autoantibody production is primarily dependent on CD4+ T cells^61^. On the other hand, SLE development in MRL-Fas/Lpr mice is attributed to a spontaneous mutation in Fas, which mediates death signal in cells. This mutation is responsible for the accelerated onset of autoimmune disease in the lupus-prone MRL strain^61,62^. Since TACI is essential for T cell-independent humoral response^41,43^, deletion of TACI in NZM mice may not have a profound effect on T cell-dependent autoantibody production in this strain. Our analysis of GC B cells, Tfh cells and Thef cells confirmed the limited role TACI plays in T cell-dependent autoantibody production in MRL-Fas/Lpr mice. Spontaneous GC formation and Tfh as well as Thef cell development are implicated in autoreactive antibody development in patients and in lupus mice^55,58,63^. Indeed, we found that MRL-Fas/Lpr mice GC B cells, Tfh, and Thef cell frequencies were markedly higher than those of wild-type MRL mice and Balb/c mice as early as 6 weeks of age. TACI deficiency appears to have contained this rapid increase in the number of GC B cells and Tfh cells until 8 weeks of age, which may have contributed to the delayed onset of autoreactive antibody development in LPR-TACI−/− mice during this early period. A more profound effect of TACI deficiency on autoreactive antibody production may have been the prevention of T cell-independent autoantibody generation, since TACI is essential for the production of antibodies against polysaccharide antigens^41,45^. The elevated anti-dsDNA serum IgM and IgG3 antibodies as early as 8 weeks of age in wild-type littermates but not in LPR-TACI−/− strains is in support of an impairment of T cell-independent humoral immune response in TACI deficient lupus mice. The delay in the emergence of the T cell-independent isotype, IgG3 in LPR-TACI−/− mice is especially noteworthy because of the well documented role of IgG3 autoantibodies in the pathogenesis of lupus nephritis^64–66^. Thus, the delay in the onset of kidney pathology and associated proteinuria in LPR-TACI−/− mice may be related to the late appearance of IgG3 antibodies in this strain. A similar conclusion was reached by Tran *et al*. who recently attributed the improved survival of TACI deficient male Nba2.Yaa mice to diminished T cell-independent autoreactive antibody production^22^.

In SLE, inflammatory signals initiated by immune complexes and cytokines lead to accumulation of M1 polarized Mϕs in the glomeruli and interstitial compartments^67^. In addition to the well-recognized M1-polarizing signals, such as IFN-γ and TNF-α^68,69^, systemically and locally elevated BAFF and APRIL^7–9,13,15,70^ may be potentiating the M1-polarization of Mϕs in SLE. We recently discovered that TACI mediates M1-polarizing signals in Mϕs of C57BL/6 mice and freshly isolated Mϕs from TACI deficient C57BL/6 mice peritoneum and skin present with phenotypic and functional characteristics of M2 Mϕs^40^. Analysis of young LPR-TACI−/− mice peritoneal Mϕs confirmed the preservation of M2-skewed phenotype in TACI deficient lupus mouse. Moreover, the kidneys of 12-week-old LPR-TACI−/− mice not only harbored fewer numbers of Mϕs than the kidneys of wild-type littermates, but their Mϕs also expressed elevated levels of molecules associated with the M2 phenotype. In concert with the significantly progressed proteinuria, kidneys of 12-week-old wild-type littermates manifested more severe histopathological changes along with the infiltration of M1-skewed Mϕs. The predominance of M2 polarized Mϕs in 12-week-old LPR-TACI−/− mice kidneys suggested that LPR-TACI−/− mice renal Mϕs may be responsible for the low proteinuria score in TACI deficient lupus mice. Supporting this hypothesis, not only did the transfer of Mϕs from age-matched LPR-TACI−/− mice halt the progression of proteinuria in 12-week-old LPR-TACI+/+ mice, but also diminished the renal histopathological changes. However, although TACI deficient lupus mice Mϕs present with M2 phenotype, they appear to succumb to inflammatory signals initiated with immune complexes because the increase in systemic autoreactive antibodies correlated with the progression of proteinuria and conversion of the renal Mϕ-phenotype to M1. Nevertheless, our study highlights a role for M2-polarized Mϕ-transfer as a therapeutic intervention to alleviate renal impairment in SLE disease^71^.

In summary, we discovered a novel function for TACI deficient Mϕs in protecting from renal disease in SLE. Together with the M2-skewed Mϕs, late onset of autoreactive antibodies contributes to the improved survival of LPR-TACI−/− mice. The primary reason for the delayed development of autoreactive antibodies appears to be the impairment in T cell-independent antibody production when TACI is absent. Profound differences in the IgM and IgG3 anti-dsDNA antibodies between LPR-TACI−/− and LPR-TACI+/+ mice during the early phases of disease is in support of this hypothesis. A meaningful contribution of the reduction in T cell-dependent antibody responses to the overall delay in autoreactive antibody production is unlikely, because only 6-week-old LPR-TACI−/− mice GC and Tfh frequencies were significantly less than the wild-type littermates. Interestingly, although M2-skewed Mϕs may be protecting LPR-TACI−/− mice from kidney pathology at early stages of disease, TACI deficient Mϕs were unable to resist the M1 polarizing cues associated with the deposition of immune complexes in the kidneys. Thus, in addition to the early ablation of T cell-independent autoreactive antibody production, M2-Mϕ mediated preservation of kidneys from inflammatory assault appears to contribute to the improved survival of LPR-TACI−/− mice.

## Methods

### Mice

MRL/MpJ-FASLPR/J (will be referred to as MRL-Fas/Lpr throughout the manuscript) and MRL/MpJ (will be referred to as MRL throughout the manuscript) mice, were purchased from The Jackson Laboratory (Bar Harbor, ME). Balb/c mice were purchased from Charles River Laboratories (Wilmington, MA). We generated LPR-TACI−/− using a backcross-intercross strategy for more than 10 generations to obtain mice with a homozygous mutation of both FAS and TACI. The progeny were screened by polymerase chain reaction (PCR) amplification of tail genomic DNA using primers for the TACI wild-type gene (sense, 5′-AGGCATGGCTATGGCATT-3′; antisense, 5′-TTCTGGGCCTTTTCTCACAG-3′) and TACI deficiency (neomycin resistance insertion; sense, 5′-AGGATCTCCTGTCATCTCACCTTGCTCCTG-3′; antisense, 5′-AAGAACTCGTCAAGAAGGCGATAGAAGGCG-3′). TACI deficient mice were also screened for 178 microsatellite markers to select the offsprings with the highest percentage of MRL genetic background. All mice were bred and housed under specific pathogen-free conditions. Only age-matched female mice were used for experiments. All mice were bred and maintained under specific pathogen-free conditions in the animal facility of US Food and Drug Administration/Center for Biologics Evaluation and Research Veterinary Services. The breeding and use of animals were approved by and carried out within accordance of the US Food and Drug Administration/Center for Biologics Evaluation and Research Institutional Animal Care and Use Committee (permit numbers 2002–31 and 2002–37). All methods were performed in accordance with the relevant guidelines and regulations.

### Evaluation of disease progression

LPR-TACI−/−, LPR-TACI+/− and LPR-TACI+/+ littermates were scored blindly for proteinuria, lymphadenopathy, and skin lesions. Cervical, brachial, and inguinal lymphadenopathy was assessed daily beginning at 6 weeks of age. Lymph nodes were palpated and the date of swollen lymph nodes in littermates was recorded. Proteinuria of female mice was measured at the ages of 3 and 20 weeks using Fisherbrand™ Urine Reagent Strips (Fisher scientific, Hampton, NH) and scored on a scale of 0–5, 0, none; 1, trace; 2, 30 mg/dl; 3, 100 mg/dl; 4, 300 mg/dl; and 5, ≥2000 mg/dl.

### Histopathological assessment of the kidneys

Kidneys were fixed in 10% buffered formalin overnight, were processed, and were embedded in paraffin. Sections were stained with Hematoxylin and Eosin (HE), Periodic Acid Schiff (PAS), Periodic acid methenamine silver (PAM) and Mason’s trichrome. All slides were digitally scanned by Hamamatsu Nanozoomer-XR digital slide scanner (Hamamatsu Photonics K. K., Japan) for semiquantitative analysis of histopathological examination. Glomerular changes, inflammatory cell infiltration, and interstitial fibrosis were evaluated and scored based on a 0–3 intervals of 0.5. 0 = no pathology, 1 = mild pathology, 2 = moderate pathology and 3 = severe pathology.

### Fluorescence microscopy

Freshly isolated mouse kidneys were flash-frozen in Tissue-Tek® OCT compound in liquid nitrogen, then stored at −80 °C until cryosectioning at −20 °C. Seven µm cryosections were cut onto SuperFrost Plus slides (Menzel-Glaser), fixed in acetone for 5 min and then air-dried at room temperature. Renal immunoglobulin (Ig) and C3 deposits were detected with anti-mouse IgG-Alexa Fluor 488 (Molecular Probes, Eugene, OR) or anti-mouse C3 (Thermofisher Science, Hampton, NH) antibodies, respectively. To detect serum autoantibodies by indirect microscopy, sera were diluted 1:50 or 1:10 in PBS and applied to HEp-2 slides and Crithidia luciliae slides (BIO-RAD, Hercules, CA), respectively. Slides were washed with PBS and stained with anti-mouse IgG-Alexa Fluor 488 (Molecular Probes, Eugene, OR) and DAPI (Sigma, St. Louis, MO). All fluorescence slides were viewed with a BX61 fluorescence microscope (Olympus, Shinjuku, Tokyo, Japan). Microscope images for each experiment were uniformly analyzed using ImageJ software (NIH, Bethesda, Maryland).

### Detection of anti-dsDNA antibodies in sera

Serum anti-dsDNA IgG antibodies were measured by ELISA. Briefly, calf thymic DNA (Sigma,St. Louis, MO) was coated on a 96-well microtiter plate (Dynatech Immulon 4 HBX; Dynatech Labs., Chantilly, VA) at 0.5 μg/ml with 0.1 M of carbonate-bicarbonate buffer (pH 9.6) overnight at 4 °C. Plates were blocked for 30 minutes at room temperature in 5% BSA in PBS, then washed with 0.05% Tween-20 in PBS. Diluted serum samples were added to plates in triplicate and incubated at 37 °C for 2 hours. Plates were washed with 0.05% Tween-20 in PBS and further incubated with HRP-conjugated goat antibodies directed against mouse total IgG, IgG2a, IgG2b, IgG3 and IgM (Southern Biotech, Birmingham, AL) for 1 hour at room temperature. Finally, plates were washed with 0.05% Tween-20 in PBS and measured at 405 nm absorbance after developing with ABTS Simple Solution (Invitrogen, Carlsbad, CA).

### Flow cytometry

Single cell suspensions of spleen were obtained by mechanic dissociation of tissue through a 40 μM cell strainer. Kidneys were minced and digested with 2 mg/ml collagenase D at 37 °C for 30 min. The dissociated cells were filtered through a 100 μM cell strainer. Red blood cells were then lysed using ACK lysing buffer (Lonza, Wallersville, MD). Spleen or kidney cells (10^7^) were stained with fluorescent anti-mouse antibodies after blocking CD16/CD32 with Fc Block (BD Biosciences, San Jose, CA). Flow cytometric analysis of mouse splenocytes was performed using the following antibodies: Pacific blue–anti-CD19, APC–anti-CD138, BV605–anti-CD3, Percp Cy5.5 anti-GL-7, Percp Cy5.5 anti-CD44, FITC anti-62L, PE anti-PSGL-1, PE-Cy7-anti–PD-1, APC anti-CXCR5, BV421 anti-CXCR4, APC anti-CD93, Percp Cy5.5 anti-IgM, APC anti-CD206, Percp Cy5.5–anti-CD86, PE anti-IL-4R, BV421 anti-F4/80, FITC anti-CD11b (All from BioLegend, San Diego, CA. FITC–peanut agglutinin (PNA) was purchased from Vector Laboratories (Burlingame, CA). Qdot605 anti-CD4 antibody was purchased form Fisher Thermo (Waltham, MA). ATTO 488 anti-BAFFR antibody was from Enzo life Science Inc. (Farmingdale, NY). BV605 anti-IgD, FITC anti-BCMA, and PE anti-TACI antibodies were from BD Biosciences\BD Pharmingen (San Jose, CA). Stained cells were analyzed using a flow cytometer (LSR II; BD) and data were analyzed using FLOWJO version 10.1 for PC (Tree Star, Ashland, OR).

### Quantitative Real-Time PCR

Total RNA was extracted from the sorted renal Mϕs using the RNeasy Mini kit (Qiagen, Germantown, MD). Two hundred nanograms of total RNA was reverse-transcribed into cDNA using random hexamers with the Taqman Reverse transcription kit (Invitrogen). The expression of M1 and M2-associated genes and GAPDH were determined using Taqman Gene Expression assays and the CFX96 Touch Real-Time System (BioRad, Hercules, CA). Relative expression values were determined by the 2−ΔCt method where samples were normalized to GAPDH expression^40^.

### Macrophage adoptive transfer experiments

Isolated F4/80+ peritoneal Mϕs were cultured (10^6^/ml) in 10 cm plates and non-adherent cells were washed away after 1 hour of culture. Mϕs were cultured for 48 hours in complete RPMI media. Cells were then washed 3 times in PBS on the plate, with the remaining cells removed using TrypLE Express Enyzme Buffer (Invitrogen). The purity was greater than 95% Mϕs (F4/80+ MHCII+ Ly6G−) as confirmed by flow cytometry. Macrophages were suspended in PBS and 5 × 10^6^ cells in 50 μl were injected i.v. into recipient mice.

### Statistical analysis

Data from groups were compared using GraphPad Prism, Version 5 software (GraphPad Software, San Diego, CA) and nonparametric testing was performed by the Mann-Whitney rank sum test for two groups and by Kruskal-Wallis one-way ANOVA on ranks for three or more groups. The log-rank test with Tukey-Kramer multiplicity adjustment was performed, using SAS 9.4, to compare survival curves and lymphadenopathy development.

## Electronic supplementary material

Supplemental information

## References

[1] Trotter K, Clark MR, Liarski VM (2016). Overview of pathophysiology and treatment of human lupus nephritis. Curr Opin Rheumatol.

[2] Tsokos GC, Lo MS, Costa Reis P, Sullivan KE (2016). New insights into the immunopathogenesis of systemic lupus erythematosus. Nat Rev Rheumatol.

[3] Fattah Z, Isenberg DA (2014). Recent developments in the treatment of patients with systemic lupus erythematosus: focusing on biologic therapies. Expert Opin Biol Ther.

[4] Hahn BH (2012). American College of Rheumatology guidelines for screening, treatment, and management of lupus nephritis. Arthritis Care Res (Hoboken).

[5] Anders HJ, Appel GB (2012). Lupus nephritis: Implications of the new ACR lupus nephritis guidelines. Nat Rev Nephrol.

[6] Schneider P (1999). BAFF, a novel ligand of the tumor necrosis factor family, stimulates B cell growth. The Journal of experimental medicine.

[7] Mackay F (1999). Mice transgenic for BAFF develop lymphocytic disorders along with autoimmune manifestations. J Exp Med.

[8] Collins CE (2006). B lymphocyte stimulator (BLyS) isoforms in systemic lupus erythematosus: disease activity correlates better with blood leukocyte BLyS mRNA levels than with plasma BLyS protein levels. Arthritis Res Ther.

[9] Stohl W (2003). B lymphocyte stimulator overexpression in patients with systemic lupus erythematosus: longitudinal observations. Arthritis Rheum.

[10] Gross JA (2000). TACI and BCMA are receptors for a TNF homologue implicated in B-cell autoimmune disease. Nature.

[11] Jacob CO (2006). Paucity of clinical disease despite serological autoimmunity and kidney pathology in lupus-prone New Zealand mixed 2328 mice deficient in BAFF. J Immunol.

[12] Cheema, G. S., Roschke, V., Hilbert, D. M. & Stohl, W. Elevated serum B lymphocyte stimulator levels in patients with systemic immune-based rheumatic diseases. *Arthritis Rheum* **44**, 1313–1319, 10.1002/1529-0131 (2001). 10.1002/1529-0131(200106)44:6<1313::AID-ART223>3.0.CO;2-S 11407690

[13] Boghdadi G, Elewa EA (2014). Increased serum APRIL differentially correlates with distinct cytokine profiles and disease activity in systemic lupus erythematosus patients. Rheumatol Int.

[14] Eilertsen GO, Nossent JC (2014). APRIL levels strongly correlate with IL-17 in systemic lupus erythematosus. Lupus.

[15] Hegazy M, Darwish H, Darweesh H, El-Shehaby A, Emad Y (2010). Raised serum level of APRIL in patients with systemic lupus erythematosus: correlations with disease activity indices. Clin Immunol.

[16] Koyama T (2005). Raised serum APRIL levels in patients with systemic lupus erythematosus. Ann Rheum Dis.

[17] Stohl W (2004). Inverse association between circulating APRIL levels and serological and clinical disease activity in patients with systemic lupus erythematosus. Ann Rheum Dis.

[18] Morel J (2009). Serum levels of tumour necrosis factor family members a proliferation-inducing ligand (APRIL) and B lymphocyte stimulator (BLyS) are inversely correlated in systemic lupus erythematosus. Ann Rheum Dis.

[19] Jacob CO (2012). Dispensability of APRIL to the development of systemic lupus erythematosus in NZM 2328 mice. Arthritis Rheum.

[20] Stein JV (2002). APRIL modulates B and T cell immunity. J Clin Invest.

[21] Huard B, Tran NL, Benkhoucha M, Manzin-Lorenzi C, Santiago-Raber ML (2012). Selective APRIL blockade delays systemic lupus erythematosus in mouse. PLoS One.

[22] Tran NL, Schneider P, Santiago-Raber ML (2017). TACI-dependent APRIL signaling maintains autoreactive B cells in a mouse model of systemic lupus erythematosus. Eur J Immunol.

[23] Ramanujam M, Davidson A (2008). BAFF blockade for systemic lupus erythematosus: will the promise be fulfilled?. Immunol Rev.

[24] Furie R (2011). A phase III, randomized, placebo-controlled study of belimumab, a monoclonal antibody that inhibits B lymphocyte stimulator, in patients with systemic lupus erythematosus. Arthritis Rheum.

[25] Navarra SV (2011). Efficacy and safety of belimumab in patients with active systemic lupus erythematosus: a randomised, placebo-controlled, phase 3 trial. Lancet.

[26] Mackay F, Schneider P (2009). Cracking the BAFF code. Nat Rev Immunol.

[27] Salazar-Camarena DC (2016). Association of BAFF, APRIL serum levels, BAFF-R, TACI and BCMA expression on peripheral B-cell subsets with clinical manifestations in systemic lupus erythematosus. Lupus.

[28] Sellam J (2007). Decreased B cell activating factor receptor expression on peripheral lymphocytes associated with increased disease activity in primary Sjogren’s syndrome and systemic lupus erythematosus. Ann Rheum Dis.

[29] Carter RH (2005). Expression and occupancy of BAFF-R on B cells in systemic lupus erythematosus. Arthritis Rheum.

[30] Zhao LD (2010). Expressions of BAFF/BAFF receptors and their correlation with disease activity in Chinese SLE patients. Lupus.

[31] Kim J, Gross JA, Dillon SR, Min JK, Elkon KB (2011). Increased BCMA expression in lupus marks activated B cells, and BCMA receptor engagement enhances the response to TLR9 stimulation. Autoimmunity.

[32] Jacob CO (2013). Development of systemic lupus erythematosus in NZM 2328 mice in the absence of any single BAFF receptor. Arthritis Rheum.

[33] Ju ZL, Shi GY, Zuo JX, Zhang JW, Jian S (2007). Unexpected development of autoimmunity in BAFF-R-mutant MRL-lpr mice. Immunology.

[34] Jiang C, Loo WM, Greenley EJ, Tung KS, Erickson LD (2011). B cell maturation antigen deficiency exacerbates lymphoproliferation and autoimmunity in murine lupus. J Immunol.

[35] Figgett WA (2015). Deleting the BAFF receptor TACI protects against systemic lupus erythematosus without extensive reduction of B cell numbers. J Autoimmun.

[36] Jacob CO (2015). Differential Development of Systemic Lupus Erythematosus in NZM 2328 Mice Deficient in Discrete Pairs of BAFF Receptors. Arthritis Rheumatol.

[37] Rottman JB, Willis CR (2010). Mouse models of systemic lupus erythematosus reveal a complex pathogenesis. Vet Pathol.

[38] Byrne JC (2012). Genetics of SLE: functional relevance for monocytes/macrophages in disease. Clin Dev Immunol.

[39] Katsiari CG, Liossis SN, Sfikakis PP (2010). The pathophysiologic role of monocytes and macrophages in systemic lupus erythematosus: a reappraisal. Semin Arthritis Rheum.

[40] Allman WR (2015). TACI deficiency leads to alternatively activated macrophage phenotype and susceptibility to Leishmania infection. Proc Natl Acad Sci USA.

[41] von Bulow GU, van Deursen JM, Bram RJ (2001). Regulation of the T-independent humoral response by TACI. Immunity.

[42] Allman WR, Liu L, Coleman AS, Akkoyunlu M (2016). MRL Strains Have a BAFFR Mutation without Functional Consequence. PLoS One.

[43] Yan M (2001). Activation and accumulation of B cells in TACI-deficient mice. Nat Immunol.

[44] Kanswal S (2011). Suppressive effect of bacterial polysaccharides on BAFF system is responsible for their poor immunogenicity. J Immunol.

[45] Kanswal S, Katsenelson N, Selvapandiyan A, Bram RJ, Akkoyunlu M (2008). Deficient TACI expression on B lymphocytes of newborn mice leads to defective Ig secretion in response to BAFF or APRIL. J Immunol.

[46] Mantchev GT, Cortesao CS, Rebrovich M, Cascalho M, Bram RJ (2007). TACI is required for efficient plasma cell differentiation in response to T-independent type 2 antigens. J Immunol.

[47] Tsuji S, Cortesao C, Bram RJ, Platt JL, Cascalho M (2011). TACI deficiency impairs sustained Blimp-1 expression in B cells decreasing long-lived plasma cells in the bone marrow. Blood.

[48] Trager J, Ward MM (2001). Mortality and causes of death in systemic lupus erythematosus. Curr Opin Rheumatol.

[49] Vlahakos D (1992). Murine monoclonal anti-DNA antibodies penetrate cells, bind to nuclei, and induce glomerular proliferation and proteinuria *in vivo*. J Am Soc Nephrol.

[50] Sugisaki T, Takase S (1991). Composition of immune deposits present in glomeruli of NZB/W F1 mice. Clin Immunol Immunopathol.

[51] Hill GS (2001). Predictive power of the second renal biopsy in lupus nephritis: significance of macrophages. Kidney Int.

[52] Iwata Y (2012). Aberrant macrophages mediate defective kidney repair that triggers nephritis in lupus-susceptible mice. J Immunol.

[53] Ou X, Xu S, Lam KP (2012). Deficiency in TNFRSF13B (TACI) expands T-follicular helper and germinal center B cells via increased ICOS-ligand expression but impairs plasma cell survival. Proc Natl Acad Sci USA.

[54] Luzina IG (2001). Spontaneous formation of germinal centers in autoimmune mice. J Leukoc Biol.

[55] Blanco P, Ueno H, Schmitt N (2016). T follicular helper (Tfh) cells in lupus: Activation and involvement in SLE pathogenesis. Eur J Immunol.

[56] Jacquemin C (2015). OX40 Ligand Contributes to Human Lupus Pathogenesis by Promoting T Follicular Helper Response. Immunity.

[57] Odegard JM (2008). ICOS-dependent extrafollicular helper T cells elicit IgG production via IL-21 in systemic autoimmunity. The Journal of experimental medicine.

[58] William J, Euler C, Christensen S, Shlomchik MJ (2002). Evolution of autoantibody responses via somatic hypermutation outside of germinal centers. Science.

[59] Seshasayee D (2003). Loss of TACI causes fatal lymphoproliferation and autoimmunity, establishing TACI as an inhibitory BLyS receptor. Immunity.

[60] Liu W (2004). Control of spontaneous B lymphocyte autoimmunity with adenovirus-encoded soluble TACI. Arthritis Rheum.

[61] Santiago-Raber ML, Laporte C, Reininger L, Izui S (2004). Genetic basis of murine lupus. Autoimmun Rev.

[62] Watanabe-Fukunaga R, Brannan CI, Copeland NG, Jenkins NA, Nagata S (1992). Lymphoproliferation disorder in mice explained by defects in Fas antigen that mediates apoptosis. Nature.

[63] Choi JY (2015). Circulating follicular helper-like T cells in systemic lupus erythematosus: association with disease activity. Arthritis Rheumatol.

[64] Greenspan NS (2012). IgG3 deficiency extends lifespan and attenuates progression of glomerulonephritis in MRL/lpr mice. Biol Direct.

[65] Jacob N, Stohl W (2010). Autoantibody-dependent and autoantibody-independent roles for B cells in systemic lupus erythematosus: past, present. and future. Autoimmunity.

[66] Takahashi S, Nose M, Sasaki J, Yamamoto T, Kyogoku M (1991). IgG3 production in MRL/lpr mice is responsible for development of lupus nephritis. J Immunol.

[67] Davidson A (2016). What is damaging the kidney in lupus nephritis?. Nat Rev Rheumatol.

[68] Murray PJ (2017). Macrophage Polarization. Annu Rev Physiol.

[69] Orme J, Mohan C (2012). Macrophage subpopulations in systemic lupus erythematosus. Discov Med.

[70] Schwarting, A. *et al*. Renal tubular epithelial cell-derived BAFF expression mediates kidney damage and correlates with activity of proliferative lupus nephritis in mouse and men. *Lupus*, 961203317717083, 10.1177/0961203317717083 (2017). 10.1177/0961203317717083 28659046

[71] Li F, Yang Y, Zhu X, Huang L, Xu J (2015). Macrophage Polarization Modulates Development of Systemic Lupus Erythematosus. Cell Physiol Biochem.

