# Supplementary material for: Delayed onset of autoreactive antibody production and M2-skewed macrophages contribute to improved survival of TACI deficient MRL-Fas/Lpr mouse
Source: Sci Rep. 2018 Jan 22;8:1308. doi: 10.1038/s41598-018-19827-8 (PMC5778001; doi:10.1038/s41598-018-19827-8)
Supplement: Supplementary file 1 — Supplemental information [file 41598_2018_19827_MOESM1_ESM.doc]

**Supplemental information**

**Delayed onset of autoreactive antibody production and M2-skewed macrophages contribute to improved survival of TACI deficient MRL-Fas/Lpr mouse**

Lunhua Liu, Windy Allman, Adam Coleman, Kazuto Takeda, Tsai-Lien Lin and Mustafa Akkoyunlu

**
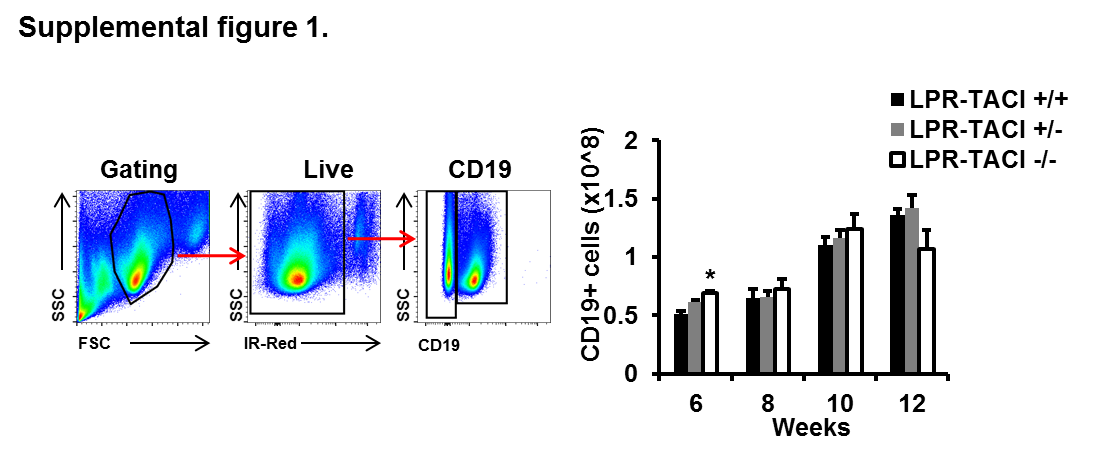
**

**Supplemental figure 1. FACS analysis of splenic B cell populations in LPR-TACI +/+, LPR-TACI +/- and LPR-TACI -/- mice.**

Flow cytometry splenic B cell gating strategy and mean of total B cells (CD19+) ± SD on weeks 6 to 12 are shown. Each group contained 5 mice. * p<0.05 indicates statistical difference between LPR-TACI +/+ and LPR-TACI -/- mice.

**
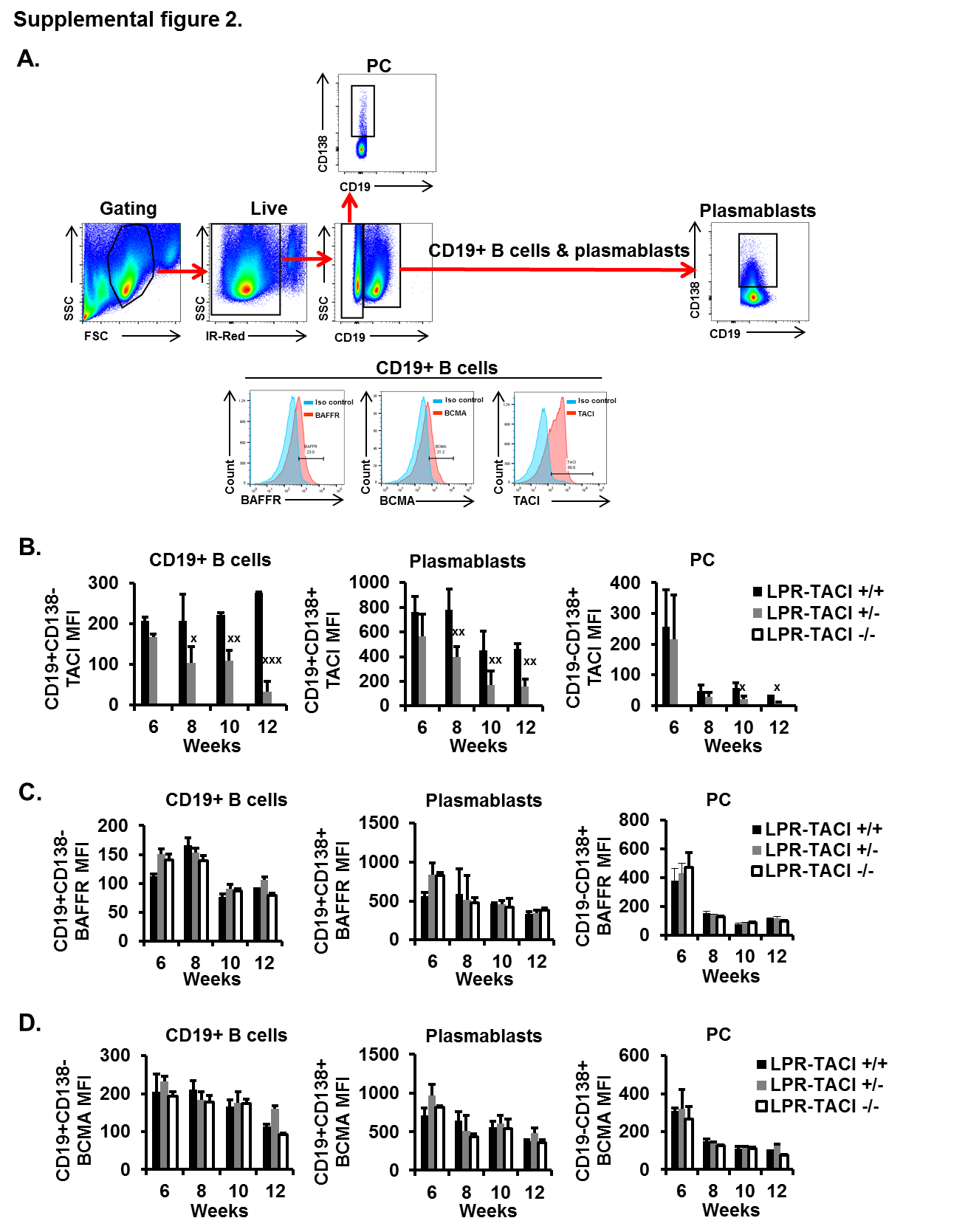
**

**Supplemental figure 2. TACI, BAFFR and BCMA expression on B cells, plasmablasts and plasma cells of LPR-TACI +/+, LPR-TACI +/- and LPR-TACI -/- mice.**

(A). Gating strategy for splenic B cells (CD19+CD138-), plasmablasts (CD19+CD138+), and plasma cells (PC, CD19-CD138+) are shown. The expression of TACI (B), BAFFR (C) and BCMA (D) on CD19+CD138- mature B cells, CD19+CD138+ plasmablasts and CD19-CD138+ plasma cells (PC) were analyzed by flow cytometry. Mean fluorescence intensity ± SD from 4 to 6 mice in each group were plotted. x p<0.05, xx p<0.01, and xxx p<0.001 indicate statistical differences between LPR-TACI +/+ and LPR-TACI +/- mice.

**
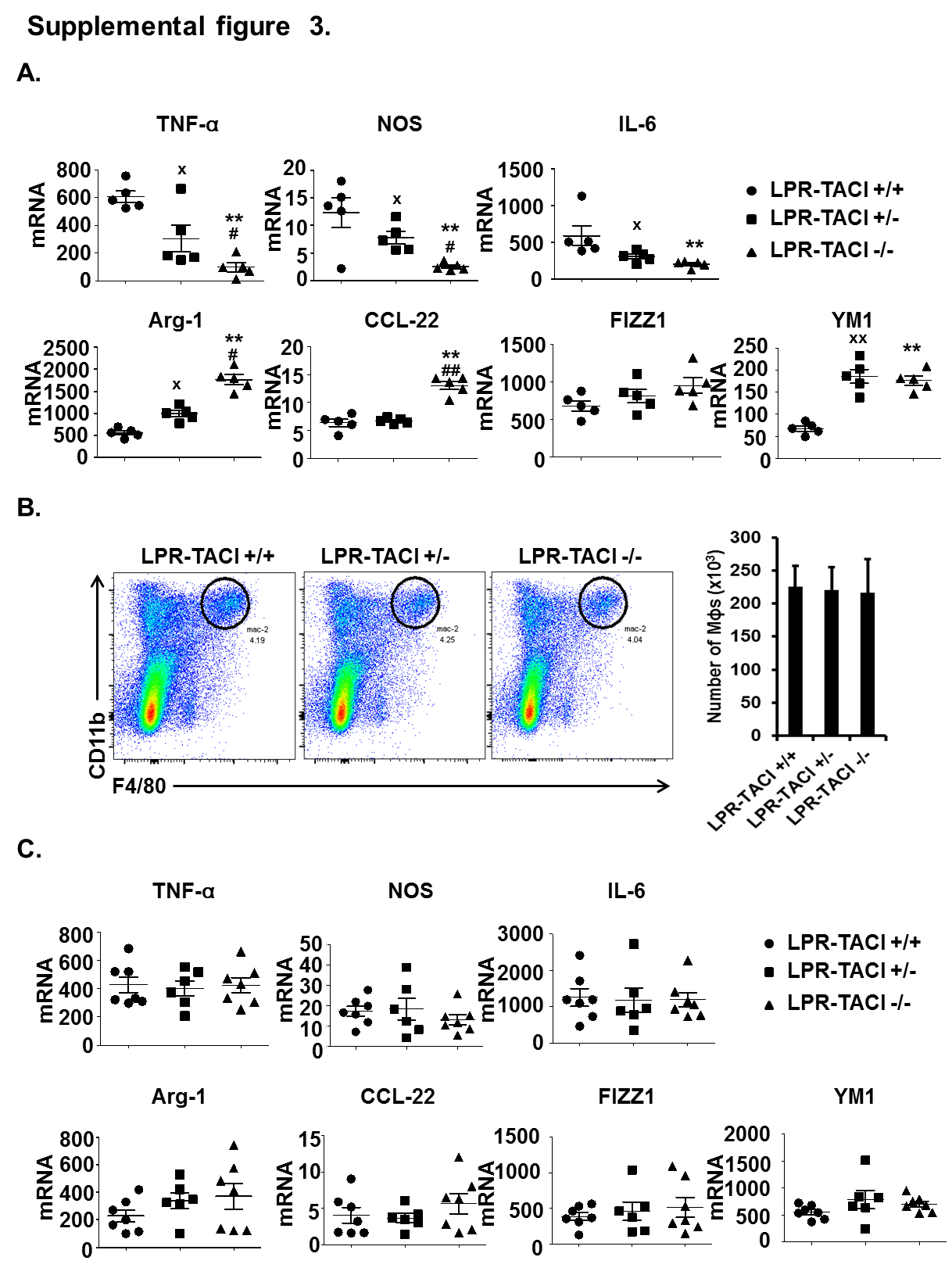
**

**Supplemental figure 3. Early time point renal Mϕ phenotype is different in LPR-TACI +/+ and LPR-TACI -/- mice.**

(A). Peritoneal Mϕs were isolated from 12-weeks old LPR-TACI +/+, LPR-TACI +/- and LPR-TACI -/- mice. Expression of M1 and M2-associated genes in the purified Mϕs was measured by Q-PCR. * p<0.05 and **p<0.01 indicate statistical differences between LPR-TACI +/+ and LPR-TACI -/- mice. # p<0.05 and ##p<0.01 indicate statistical differences between LPR-TACI +/- and LPR-TACI -/- mice. x p<0.05 indicates statistical differences between LPR-TACI +/+ and LPR-TACI +/- mice. (B). Representative pseudocolor dot plots of renal Mϕs from 26-week old LPR-TACI +/+, LPR-TACI +/- and LPR-TACI -/- mice. Kidneys from 26-week old mice were first minced and then digested with collagenase. The fraction containing immune cells were isolated after Percoll gradient centrifugation. Following the exclusion of dead cells, Mϕs were gated as F4/80 and CD11b positive cells and quantified. (C). Renal Mϕs were sorted from 26-week old mice. Expression of M1 and M2-associated genes in the sorted cells was assessed by Q-PCR.

**
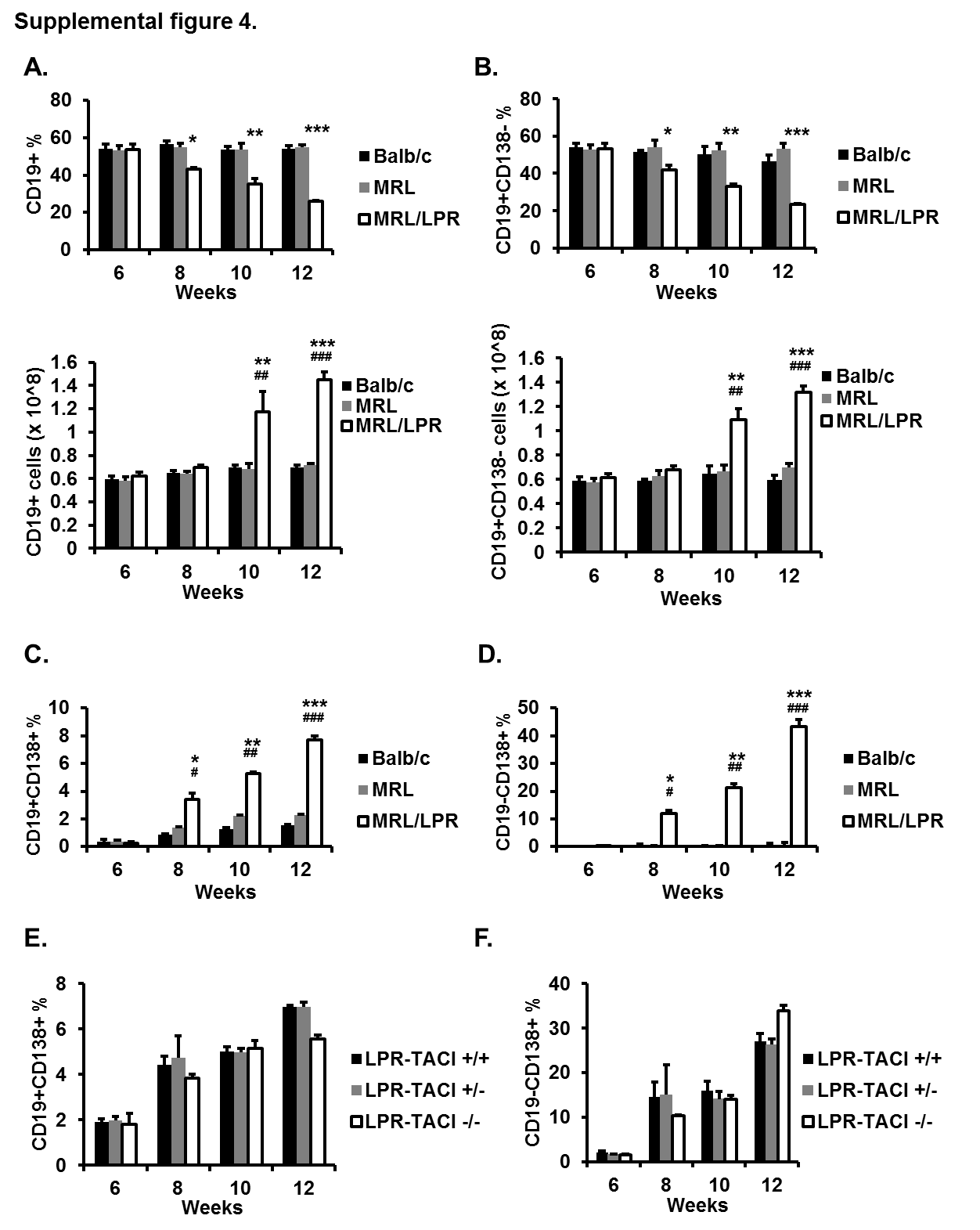
**

**Supplemental figure 4. Plasmablast and plasma cell numbers increase with age in MRL/LPR as well as in LPR-TACI -/- mouse and its litter mates.**

Percentages and numbers of total CD19+ (A) and CD19+CD138- mature B cells (B) in spleens of 6 to 12 weeks old Balb/c, MRL and MRL/LPR mice were quantified in flow cytometry. Percentages of CD19+CD138+ plasmablasts (C), and CD19-CD138+ plasma cells (D) in spleens of 6 to 12 weeks old Balb/c, MRL and MRL/LPR mice were quantified in flow cytometry. Mean percentages ± SD from 5 mice in each group were plotted. Percentages of total CD19+CD138+ plasmablast (E), and CD19-CD138+ plasma cells (F) in spleens of 6 to 12 weeks old LPR-TACI +/+, LPR-TACI +/- and LPR-TACI -/- mice were quantified in flow cytometry. * p<0.05, ** p<0.01 and ***p<0.001 indicate statistical differences between Balb/c and MRL/LPR mice. # p<0.05, ## p<0.01 and ### p<0.001 indicate statistical differences between MRL and MRL/LPR mice.

**
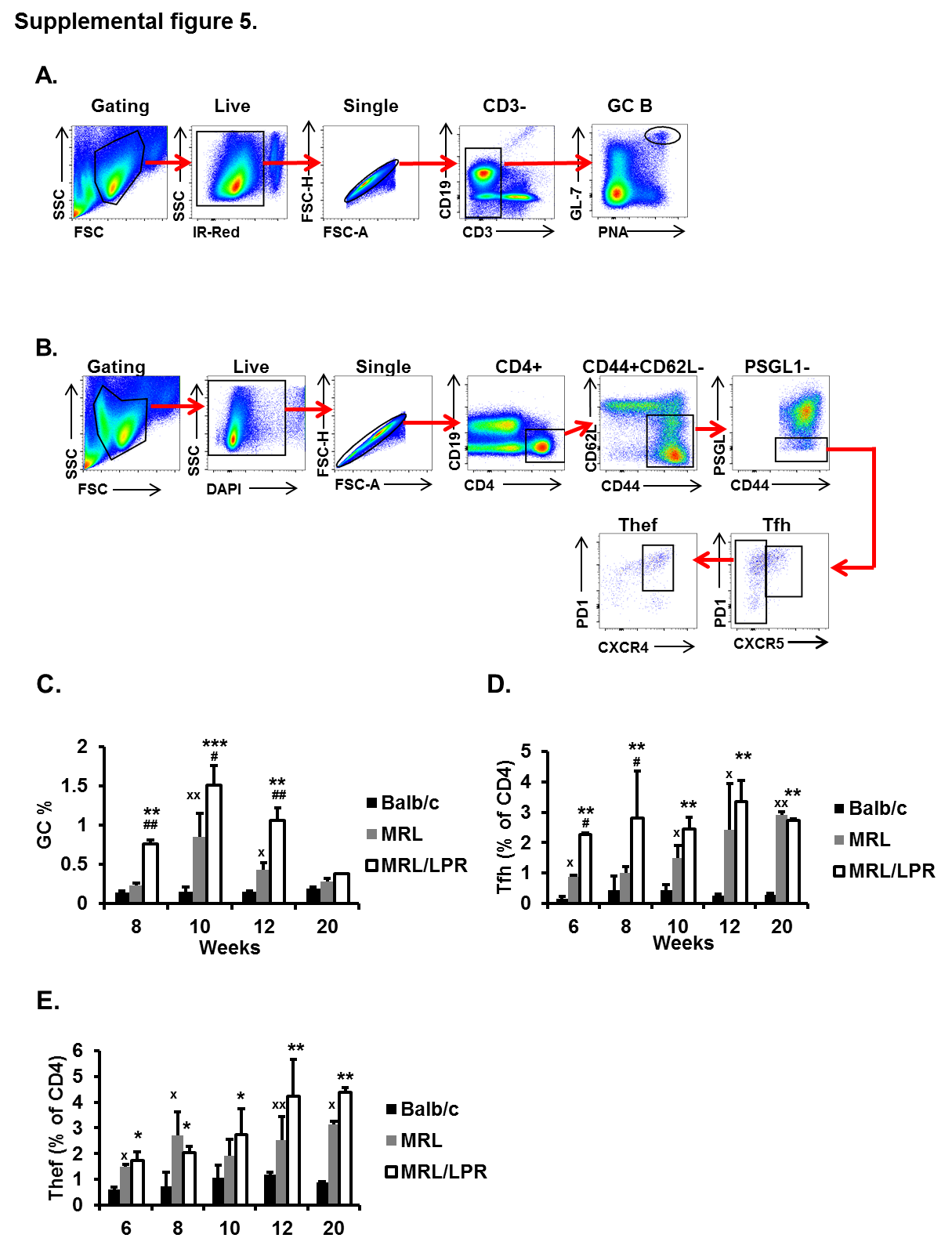
**

**Supplemental figure 5. Kinetics of spontaneous splenic GC-, Tfh- and Thef-cell formation in MRL/LPR, MRL and Balb/c mice.**

(A). Gating strategy for spontaneously formed GC B cells in flow cytometry. Lymphocyte populations were gated based on forward and side scatter, and then CD19+CD3-cell populations were gated on live singlet cells. Spontaneous GC populations were determined by GL-7 and PNA positive staining. (B). Gating strategy for Tfh and Thef cells in flow cytometry. Lymphocyte populations were gated based on forward and side scatter, and then live and single cells were selected. After pre-gating CD4+ cells, Tfh cells were gated as CXCR4-CXCR5+PD1+PSGL1loCD62L-CD44+cells, while Thef cells were gated as CXCR4+CXCR5-PD1+PSGL1loCD62L-CD44+cells. (C). Formation of CD19+CD3-GL7+PNA+ GC in splenocytes of 8 to 20-week old Balb/c, MRL and MRL/LPR mice was quantified in flow cytometry. Mean percentages ± SD from 5 mice in each group were plotted. (D). Percentages of CXCR4-CXCR5+PD1+PSGL1loCD62L-CD44+ Tfh cells among CD4+ splenocytes were measured in flow cytometry. Mean percentage ± SD from 5 mice in each group were plotted. (E). Percentages of CXCR4+CXCR5-PD1+PSGL1loCD62L-CD44+ Thef cells among CD4+ splenocytes was measured in FACS. Mean percentages ± SD from 5 mice in each group were plotted. * p<0.05, ** p<0.01 and ***p<0.001 indicate statistical differences between Balb/c and MRL/LPR mice. # p<0.05 and ## p<0.01 indicate statistical differences between MRL and MRL/LPR mice. x p<0.05, and xx p<0.01 indicate statistical differences between Balb/c and MRL mice.
